# Generative AI as a cognitive support tool in early-stage design ideation: A human–AI co-ideation experiment

**DOI:** 10.1371/journal.pone.0358542

**Published:** 2026-09-17

**Authors:** Yu-Ju Lin

**Affiliations:** Department of Commercial Design and Management, National Taipei University of Business, Taipei, Taiwan; Tel Aviv University, ISRAEL

## Abstract

Generative artificial intelligence is increasingly used in creative practice, but controlled evidence on its role in early-stage design ideation remains limited. This study examined whether a combined text-and-image AI-assisted workflow differed from human-only brainstorming in expert-rated ideation outputs, preliminary design refinement, and subjective task experience. A counterbalanced within-subjects experiment was conducted with 32 undergraduate design students, each of whom completed both conditions using the same design brief. Ideation outputs were evaluated by three expert raters across six task-specific analytic criteria, participant-matched concept sheets were assessed across five design attributes, and participants completed a task-specific post-task questionnaire after each condition. After Holm–Bonferroni correction within each outcome family, AI-assisted outputs received higher ratings for problem framing, mechanism, creator perspective, and evaluator perspective, whereas human-only outputs received higher ratings for innovation and proposed solution strategies. No statistically robust condition differences were found for the five design attributes. Participants reported higher ratings for perceived cognitive processing, self-rated ideation skills, and affective response in the AI-assisted condition. These findings indicate different profiles of evaluated ideation output rather than a uniform advantage for either condition. Perceived support in the AI-assisted workflow did not translate into a uniform advantage across originality-related ratings or into superior expert-rated preliminary design refinement.

## Introduction

Recent advances in large language models and diffusion-based image generation have expanded the use of generative artificial intelligence in creative work. These systems are increasingly used to retrieve information, produce finished content, and generate textual, visual, and conceptual materials that support early idea development [1–3]. As a result, generative artificial intelligence has attracted growing attention as a possible support mechanism within human creative processes rather than merely as an output-producing technology.

In design cognition, early-stage ideation is a critical phase in which designers interpret a brief, frame problems, generate alternatives, and gradually develop provisional concepts into more coherent directions. Divergent thinking plays an important role in this stage because open-ended design tasks require the exploration of multiple possibilities rather than the immediate selection of a single solution [4,5]. However, early-stage ideation is also vulnerable to several constraints, including fixation on familiar examples, premature convergence, and difficulty sustaining broad conceptual exploration when the task remains ambiguous [6–8]. These challenges may be especially salient in educational design contexts, where learners are still developing strategies for exploratory thinking and concept development [9–11].

Prior research on creativity support tools has described interactive systems that help users externalize ideas, explore alternatives, and sustain creative search [3,12–16]. From this perspective, generative artificial intelligence may be understood as a contemporary form of cognitive support in design work. Rather than replacing the designer, it may provide prompts, variations, and alternative representations that extend exploratory activity during ideation [17–25]. Related work has also suggested that AI and other digital support tools may assist exploratory thinking by helping users generate provisional options, compare alternatives, and extend creative search processes [26,27]. At the same time, the value of such support cannot be assumed. AI-generated suggestions may broaden conceptual search in some situations, but they may also encourage superficial acceptance, reduce reflective judgment, or create new forms of fixation when machine-generated outputs are treated as authoritative starting points rather than provisional resources [28–32].

Although discussion of human–AI collaboration in creative practice has increased rapidly, controlled evidence on how AI support affects the ideation process itself remains limited [33–37]. Existing studies have often focused on artifact-level outcomes such as novelty, preference, productivity, or aesthetic evaluation [12,38–40]. Fewer studies have examined whether AI assistance changes the functional structure of early-stage ideation, whether it benefits some aspects of ideation output more than others, or whether its influence extends beyond design outputs to participants’ subjective experience during the task [12,38–40]. This gap is important because early-stage design ideation involves the generation, organization, comparison, and evaluation of emerging design possibilities within a specific design context [9,11,41].

To address this gap, the present study examines a combined text-and-image generative AI workflow as a potential cognitive support resource through a controlled comparison with human-only brainstorming among undergraduate design students with prior design training and relatively limited experience in structured AI-supported ideation [24,25,42–44]. A counterbalanced within-subjects design was used so that each participant completed both conditions using the same design brief. The study examined three linked but non-equivalent outcome domains: task-specific expert ratings of ideation output, expert ratings of participant-matched concept sheets, and participants’ subjective task experience. Its contribution lies in specifying how condition-related differences are distributed across task-specific ideation criteria and in examining whether those differences extend to preliminary design refinement and perceived task experience. The six ideation criteria were used as analytic categories for the present task and were not proposed as a newly validated psychometric scale.

### Literature review, research questions, and hypothesis development

#### Design cognition: divergent thinking, fixation, and support for early-stage exploration.

Creative ideation occupies a central position in design cognition because it enables designers to explore multiple possible directions before selecting and refining a final solution. In early-stage design exploration, divergent thinking is particularly important because it supports the generation of varied responses, alternative associations, and expanded conceptual possibilities in response to open-ended design briefs [9–11]. At the same time, ideation is not solely a generative process. Contemporary perspectives in design cognition emphasize that early-stage design involves both generative exploration and analytical evaluation, requiring designers to iteratively construct, compare, and refine emerging ideas [41].

A persistent challenge in this process is design fixation. Design fixation refers to the tendency to adhere too closely to prior examples, familiar solutions, or salient features encountered during concept generation [6]. Empirical studies have shown that exposure to examples may constrain conceptual output, reduce originality, and narrow the range of alternatives considered [8,45]. Subsequent research further suggests that fixation is not a single phenomenon but a broader set of constraints shaped by the timing, form, and specificity of available stimuli [46–49]. These effects are particularly relevant in early-stage design tasks, where maintaining exploratory breadth is critical for effective problem framing and idea development.

To address these constraints, prior research has proposed creativity support tools as systems that assist users in externalizing ideas, exploring alternatives, and sustaining creative search processes [3,13–16]. Such tools are especially valuable in early-stage ideation contexts, where designers must repeatedly expand, reorganize, and reinterpret emerging concepts under conditions of uncertainty.

### Human–AI co-ideation: collaboration and differentiated roles

Generative artificial intelligence, including large language models and diffusion-based image generation systems, has expanded the possibilities of human–AI collaboration in creative work by enabling systems to produce textual, visual, and conceptual materials in response to human prompts [50–52]. In this context, AI is increasingly viewed as a collaborative resource that supports ideation, problem framing, and concept development [33–35].

Research on human–AI co-creation suggests that AI may support ideation in several ways. First, generative systems can rapidly produce multiple representations and alternative framings, thereby expanding the range of possibilities available during early exploration [17,21]. Second, AI-generated outputs may function as external stimuli that encourage users to reconsider assumptions and pursue additional directions [22,23]. Third, in educational and design contexts, AI-supported systems have been shown to facilitate iterative exploration and reduce barriers to initiating ideation [24,25].

However, the effects of human–AI collaboration are not uniformly positive. The effectiveness of AI support depends on factors such as task structure, user expertise, system transparency, and the degree to which human control is maintained in decision-making [36,37]. In addition, AI-generated suggestions may introduce new forms of fixation or reduce reflective judgment if users accept outputs uncritically [30–32]. These observations suggest that human–AI co-ideation should be examined as a differentiated process in which the roles of human participants and AI tools may vary across task stages and contexts.

### AI-assisted ideation as a cognitive support mechanism

From the perspective of design cognition, generative AI may be interpreted as a cognitive support tool that assists exploratory activity during early-stage ideation. Unlike traditional creativity support tools, generative AI can produce both textual and visual outputs, enabling users to access multiple representations and alternative concept directions in real time [17,25,53,54]. This capability may be particularly relevant in early-stage design tasks, where designers must extend conceptual search, reframe problem structures, and generate multiple candidate solutions.

Empirical research suggests that AI-assisted ideation can expand conceptual search spaces and facilitate exploratory thinking, particularly in contexts where initial idea generation is constrained [42–44]. At the same time, current evidence does not support the claim that AI uniformly improves design quality or creative performance across all dimensions. Instead, the effects of AI appear to be selective and context-dependent. When used as a source of provisional alternatives and exploratory stimuli, AI may support idea expansion and structural organization. When used uncritically, it may reduce independent evaluation and limit deeper engagement with design decisions [31,32].

These findings suggest that the role of AI in design ideation is best understood as a conditional support mechanism rather than as a universally superior approach. Accordingly, its effects should be examined at the level of specific ideation functions rather than as a single global outcome.

### Research questions and hypothesis development

Prior research indicates that the effects of generative AI in creative work are conditional and task-dependent rather than uniformly positive [36,37,55,56]. Generative systems may support early-stage ideation by providing alternative representations, provisional concept directions, and external stimuli for problem exploration. However, the value of these outputs depends on how designers interpret, evaluate, and transform them, and prior studies have reported both exploratory benefits and potential limitations related to originality, fixation, critical judgment, and convergence around plausible AI-generated suggestions [18,31,32,42–44,53,54].

Accordingly, the present study distinguishes among three outcome domains rather than treating creativity as a single global construct: ideation output, preliminary design refinement, and subjective task experience. This distinction allows condition-related differences to be examined at different levels of early-stage design work without presuming that AI assistance produces a generalized advantage across all outcomes.

### RQ1: Ideation output

Early-stage design ideation involves several distinguishable criteria for evaluating ideation output, including problem framing, mechanism, creator perspective, evaluator perspective, innovation, and proposed solution strategies. Generative AI may support problem exploration and conceptual expansion by supplying multiple representations and provisional directions. At the same time, AI-generated suggestions may also encourage convergence or reduce originality when users rely heavily on plausible machine-generated alternatives [18,31,32,54].

Although prior research provides reasons to expect that AI assistance may influence these criteria differently, the existing evidence does not establish sufficiently consistent directional predictions for each of the six criteria. The study therefore addresses the following research question:

RQ1: How do human-only and AI-assisted ideation differ in expert ratings of problem framing, mechanism, creator perspective, evaluator perspective, innovation, and proposed solution strategies?

### RQ2: Preliminary design refinement

Generative AI may contribute to the elaboration of initial ideas by providing textual expansions and visual alternatives. However, preliminary design refinement also depends on aesthetic judgment, contextual interpretation, material understanding, stylistic coherence, and the designer’s ability to translate provisional concepts into intentional visual outcomes.

Existing research suggests that AI-assisted outputs may receive more favorable evaluations on selected attributes under particular conditions, but it does not provide consistent evidence of a general advantage across all aspects of design quality [57,58]. It is therefore more appropriate to examine design refinement without assuming a directional superiority of either condition:

RQ2: Do participant-matched concept sheets produced under the human-only and AI-assisted conditions differ in expert ratings of form, material, color, pattern, and style?

### H1: Subjective task experience

In addition to evaluated outputs, AI-assisted ideation may influence participants’ subjective experience during the design process. Generative AI can provide immediate textual and visual responses, alternative perspectives, and iterative support, which may increase perceived assistance in organizing, generating, and refining ideas. Prior studies have reported that AI-supported ideation can influence perceived cognitive support, confidence, engagement, and ideation efficiency [42–44].

At the same time, positive subjective experience should not be interpreted as evidence of objectively stronger creative performance or deeper cognitive processing [32,59–61]. Nevertheless, the existing evidence provides a clearer basis for a directional prediction regarding participants’ perceived task experience.

H1: Participants will report more positive subjective task experiences in the AI-assisted condition than in the human-only condition, including higher ratings for perceived cognitive processing, self-rated ideation skills, and affective response.

## Methods

### Study design

This study employed a counterbalanced within-subjects experimental design to examine the role of generative artificial intelligence as a cognitive support tool in early-stage design ideation. Each participant completed two ideation conditions: a human-only brainstorming condition and an AI-assisted ideation condition. Both conditions were conducted using the same design brief, the same ideation time limit, and the same output requirements.

The within-subjects design was selected to reduce inter-individual variability associated with differences in prior design ability, ideation strategies, and visual communication skills, while also increasing statistical power by allowing each participant to serve as their own control. This approach improved sensitivity to condition-related effects while maintaining a manageable sample size in a controlled classroom-based setting.

To reduce potential order effects, participants were assigned to two equal counterbalanced sequence groups: (1) human-only ideation followed by AI-assisted ideation, or (2) AI-assisted ideation followed by human-only ideation. Participants P01-P16 were assigned to the human-first sequence, whereas participants P17-P32 were assigned to the AI-first sequence. The assigned sequence was maintained throughout the study. Sequence allocation was balanced but not randomized; therefore, unmeasured differences between the two sequence groups cannot be fully excluded. Exploratory sequence analyses compared the two sequence groups on participant-level condition difference scores for ideation performance, design attribute ratings, and subjective response measures. For each outcome, a difference score was calculated by subtracting the human-only score from the AI-assisted score, and the resulting scores were compared between sequence groups using independent-samples *t*-tests after checking normality and homogeneity assumptions. These analyses addressed systematic condition-order differences; however, they could not rule out carryover arising from repeated exposure to the same design brief. Task understanding, concepts, or visual strategies developed in the first condition may therefore have influenced work in the second condition. This distinction is acknowledged explicitly in the Limitations and future research section.

Normality of the participant-level condition-difference scores and homogeneity of variance were examined. Because several sequence-group distributions departed from normality, Mann–Whitney *U* tests were additionally conducted as sensitivity analyses. These analyses yielded the same substantive conclusion as the independent-samples *t*-tests.

### Participants

A total of 32 undergraduate students were recruited from a design department at a university in Taiwan. The mean age of the participants was 20.8 years. Most participants were third-year students and had received prior training in visual communication and digital design. Participants were familiar with commonly used design software, including Adobe Photoshop and Adobe Illustrator, and reported basic exposure to generative AI tools such as Midjourney and ChatGPT. However, they were considered relatively inexperienced in using generative AI for structured design ideation tasks. Participation was voluntary. No monetary compensation or academic credit was provided. This sample was considered appropriate for examining early-stage ideation processes in a design education context, where participants possess foundational design knowledge but limited experience in AI-assisted ideation. No direct personal identifiers were collected in the analytic dataset.

### Experimental context and task

The experiment was conducted in a structured studio-based classroom environment to ensure consistency across participants. All participants completed the same culturally situated product-design task focused on developing a temple-inspired cultural product for Gen Z users in Taiwan. The task required participants to identify user needs, interpret contextual constraints, and generate concept proposals with potential relevance to cultural and creative applications. The design brief included basic contextual information, target users, and functional requirements, while maintaining sufficient openness to allow divergent exploration. Participants were required to produce ideation outputs and subsequently develop one concept from each condition into a standardized concept sheet. Each concept sheet included a title, a short concept description, and a visual representation. All concept sheets were reformatted to a common template and anonymized prior to evaluation.

All participants completed the same design task: “Design a temple-inspired cultural product for Gen Z users in Taiwan, integrating local cultural elements, interactivity, and contemporary visual aesthetics.” The task required participants to interpret the cultural context and user group, generate multiple possible concept directions, and select one direction for preliminary development.

### Procedure

The two experimental conditions were conducted on separate regular class dates according to the course schedule. Each condition followed the same three-stage task structure: (1) problem framing, (2) initial idea exploration, and (3) selection and finalization of one concept direction. Each stage lasted 20 minutes, resulting in a total ideation duration of 60 minutes per condition. Participants documented their work throughout the three stages.

Following the 60-minute ideation task, participants selected one concept from the corresponding condition and assembled it into a standardized concept sheet. Each concept sheet included a title, a brief concept description, and a visual representation. No separate standardized time limit was imposed for concept-sheet assembly; however, the instructions and required information structure were identical across conditions. In the AI-assisted condition, participants were permitted to retain selected Midjourney-generated visual material produced during the preceding ideation process, but no additional AI-generated content was produced during concept-sheet assembly.

Each participant produced one concept sheet under each condition, yielding 32 participant-matched pairs of concept sheets (64 concept sheets in total) for the design-refinement evaluation. Immediately after completing the concept-sheet assembly for each condition, participants completed the task-specific subjective-response questionnaire for that condition. The same questionnaire structure was used for both conditions, with the wording referring respectively to the human-only or AI-assisted ideation process.

The ideation-process materials generated during each condition were retained for evaluation. These materials documented the available progression from initial problem interpretation and idea generation to concept comparison, selection, and preliminary development. Before expert evaluation, the final concept sheets were reformatted into a common layout and anonymized with respect to participant identity. The complete design brief, stage-specific instructions, submission requirements, tool-role mapping, and structured prompt guidance are provided in S4 Appendix.

### Human-only ideation condition

In the human-only condition, participants completed the design task without the use of generative AI tools or other external ideation systems. The condition comprised three consecutive stages: (1) problem framing, (2) initial idea exploration, and (3) selection and finalization of one concept direction. Each stage lasted 20 minutes, resulting in a total task duration of 60 minutes for the condition.

During the problem-framing stage, participants interpreted the shared design brief, identified relevant contextual and user considerations, and organized their initial understanding through keywords and mind maps. During the initial idea-exploration stage, they generated multiple possible directions using rough sketches, written annotations, associative thinking, and self-generated concept notes. During the final stage, participants compared the emerging alternatives, selected the most promising direction, and further articulated the selected concept through sketches and explanatory notes.

Participants worked individually and relied on their own knowledge, design experience, and internal ideation strategies throughout all three stages. The design brief, classroom setting, stage durations, and required outputs were equivalent to those used in the AI-assisted condition.

### AI-assisted ideation condition

In the AI-assisted condition, participants used a combined text-and-image generative AI workflow to support conceptual and visual exploration. ChatGPT-4o was available for text-based problem reframing, idea expansion, and concept development, whereas Midjourney v6 was available for visual exploration and concept visualization. The publicly available web-based versions of both systems were used during the study period. No API access, parameter customization, temperature adjustment, or researcher-imposed system settings were used. Because both tools were incorporated within the same experimental condition, the study estimates the effect of the combined multimodal workflow and does not permit causal separation of language-based reframing, image-based stimulation, or their interaction.

Before completing the AI-assisted condition, participants received a 30-minute standardized training session introducing the operational functions of ChatGPT-4o and Midjourney v6, prompt-based interaction procedures, and the experimental workflow. Participants were introduced to a structured prompt framework specifying the target user or subject, design objective, temporal and geographical context, interaction approach, and intended output format. This framework was used to provide contextual and representational detail while allowing participants to revise and extend their prompts according to their own design directions.

The AI-assisted condition comprised three consecutive stages: (1) problem framing, (2) initial idea exploration, and (3) selection and finalization of one concept direction. Each stage lasted 20 minutes, resulting in a total task duration of 60 minutes. During the problem-framing stage, participants used ChatGPT-4o to interpret the shared brief, examine user and contextual considerations, and generate alternative conceptual directions. During the initial idea-exploration stage, participants iteratively refined textual prompts and used ChatGPT-4o and Midjourney v6 to develop and visualize multiple possible concepts. During the final stage, participants compared the emerging alternatives, selected the most promising concept direction, and further articulated the selected proposal using the textual and visual materials generated during the AI-assisted ideation process.

Prompt iteration was unrestricted throughout the three ideation stages. No fixed Midjourney style presets or researcher-imposed output-selection rules were applied. All participants worked individually using the same design brief, three-stage task structure, 20-minute allocation for each stage, classroom setting, and required outputs as in the human-only condition.

Although ChatGPT-4o and Midjourney v6 were available within the same experimental condition, their intended roles differed across the workflow. ChatGPT-4o was used primarily for interpreting and reframing the design brief, generating textual concept directions, expanding functional or cultural considerations, and refining prompts. Midjourney v6 was used primarily for translating selected textual directions into preliminary visual alternatives and supporting visual comparison. Participants remained responsible for revising prompts, comparing generated alternatives, selecting a concept direction, and determining how the generated materials were incorporated into the proposal.

These functional distinctions describe the intended workflow but do not provide separate causal estimates for the two tools. Because participants could move iteratively between textual and visual generation and because complete tool-specific interaction logs were not collected in a standardized form, the contributions of ChatGPT-4o, Midjourney v6, and their interaction could not be analyzed independently.

Following the 60-minute ideation process, participants assembled the selected concept into a standardized concept sheet. No new AI-generated content was produced during this subsequent concept-sheet assembly stage; however, selected Midjourney-generated images produced during the ideation process were retained as visual representations in the final concept sheets. Representative prompt templates and an illustrative prompt-refinement sequence are provided in S4 Appendix. These materials support procedural replication of the task structure, timing, intended tool roles, and prompt framework. Because generative AI systems are stochastic and may change over time, exact regeneration of identical textual or visual outputs should not be expected. The materials do not constitute complete participant interaction logs and do not permit causal separation of the contributions of ChatGPT-4o, Midjourney v6, or their interaction.

**Fig 1:**
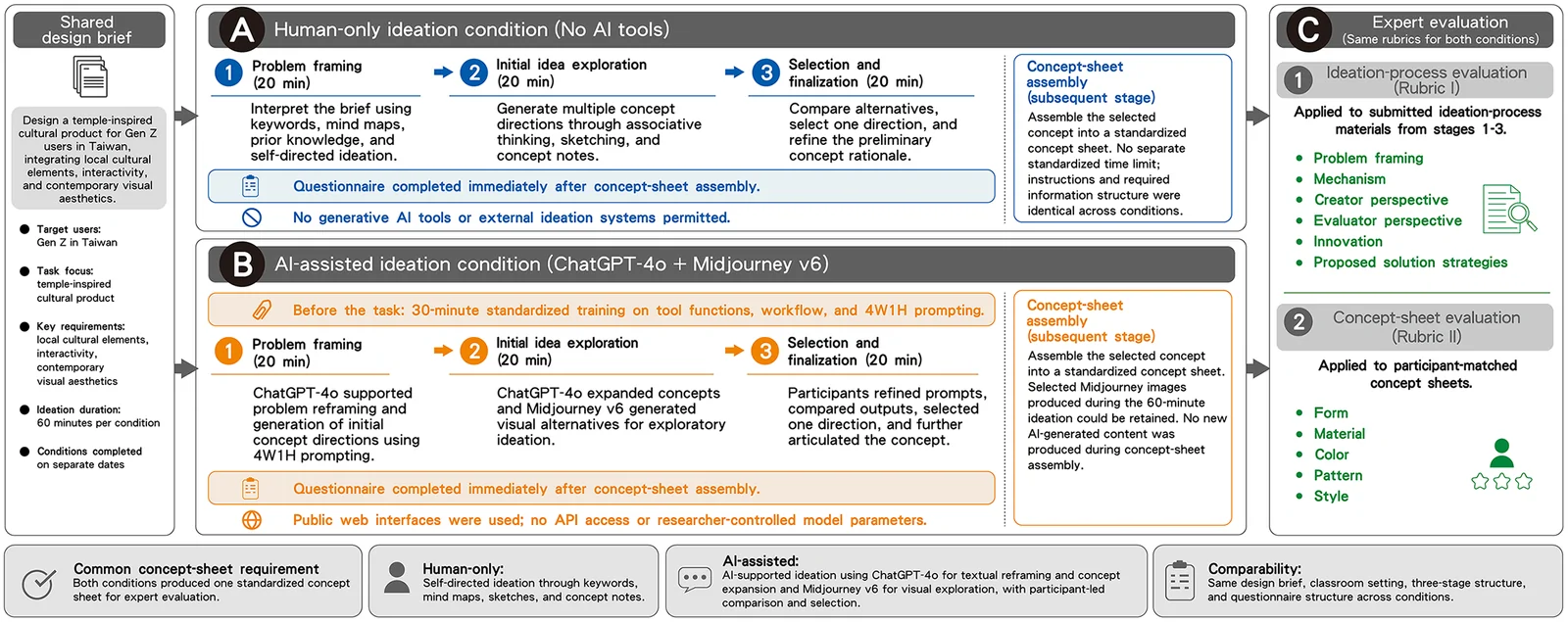
Complete experimental comparison of human-only and AI-assisted ideation workflows.

A complete comparison of the human-only and AI-assisted ideation workflows is presented in Fig 1.

### Expert evaluation

Design outputs were evaluated using a structured independent expert-assessment approach. Three independent raters conducted the evaluation: two senior design experts with more than ten years of experience and one industry practitioner with experience in conceptual product design. Each rater independently evaluated the complete set of submitted materials.

For each participant and experimental condition, the raters reviewed the full set of ideation-process materials available from the study. In the human-only condition, these materials included mind maps, keyword lists, rough sketches, self-generated concept notes, and records of concept development and refinement. In the AI-assisted condition, the materials included available participant prompts, ChatGPT-generated textual responses, prompt-refinement materials, Midjourney-generated visual alternatives, records of concept selection and development, and the resulting concept proposal. These materials provided observable evidence of how ideas were framed, expanded, compared, selected, and translated into preliminary design directions.

The evaluation was conducted in two separate rating stages. In the first stage, the raters reviewed the submitted ideation-process materials and evaluated six task-specific criteria: problem framing, mechanism, creator perspective, evaluator perspective, innovation, and proposed solution strategies (Table 1). These ratings were based on observable evidence across the submitted process materials rather than solely on the final design outcome. In the second stage, the raters evaluated the final concept sheets produced under the two conditions across five visible design attributes: form, material, color, pattern, and style (Table 2).

**Table 1:** Task-specific analytic criteria used for ideation evaluation.

Criterion	Brief definition
Problem framing	Clarity in defining and focusing the design problem
Mechanism	Breadth and diversity of ideation strategies
Creator perspective	Distinctiveness and originality of the participant’s conceptualization as expressed in the proposal
Evaluator perspective	Visible comparison, selection, and justification of ideas
Innovation	Breakthrough potential and relevance to user or market needs
Proposed solution strategies	Clarity, structure, and feasibility of translating ideas into actionable proposals

**Table 2:** Design attributes used for participant-matched concept-sheet evaluation.

Attribute	Brief definition
Form	Structural clarity, proportion, and visual coherence
Material	Suitability and expressive quality of material choices
Color	Integration and appropriateness of color application
Pattern	Fit and visual attractiveness of graphic elements
Style	Consistency and recognizability of overall design style

Each final concept sheet included a title, a brief concept description, and a visual representation and was reformatted into a common layout before evaluation. In the AI-assisted condition, selected visual material generated with Midjourney during the 60-minute ideation process was retained in the final concept sheet. No additional AI-generated content was produced during the subsequent concept-sheet assembly stage.

All participant identifiers and explicit condition labels were removed before evaluation. However, the original textual and visual characteristics of the submitted materials were retained to preserve evidence of the ideation process. Consequently, the evaluation was blinded to participant identity but not fully blinded to experimental condition. The raters could plausibly recognize the conditions from differences in representational format, such as handwritten sketches and notes in the human-only condition and generated text or imagery in the AI-assisted condition. Condition recognition, visual fidelity, and presentation-related expectations may therefore have influenced the ratings and are acknowledged as methodological limitations.

Before formal evaluation, all raters received the complete rubric, five-level scoring anchors, and standardized written instructions. The operational definitions and intended application of each criterion were explained before scoring commenced. The raters were instructed to base their judgments on the conceptual content, design relevance, coherence, originality-related qualities, and intended design characteristics evidenced in the submitted materials. Photorealism, rendering sophistication, image resolution, and technical image-generation quality were not treated as independent scoring criteria or as proxies for design quality.

**Fig 2:**
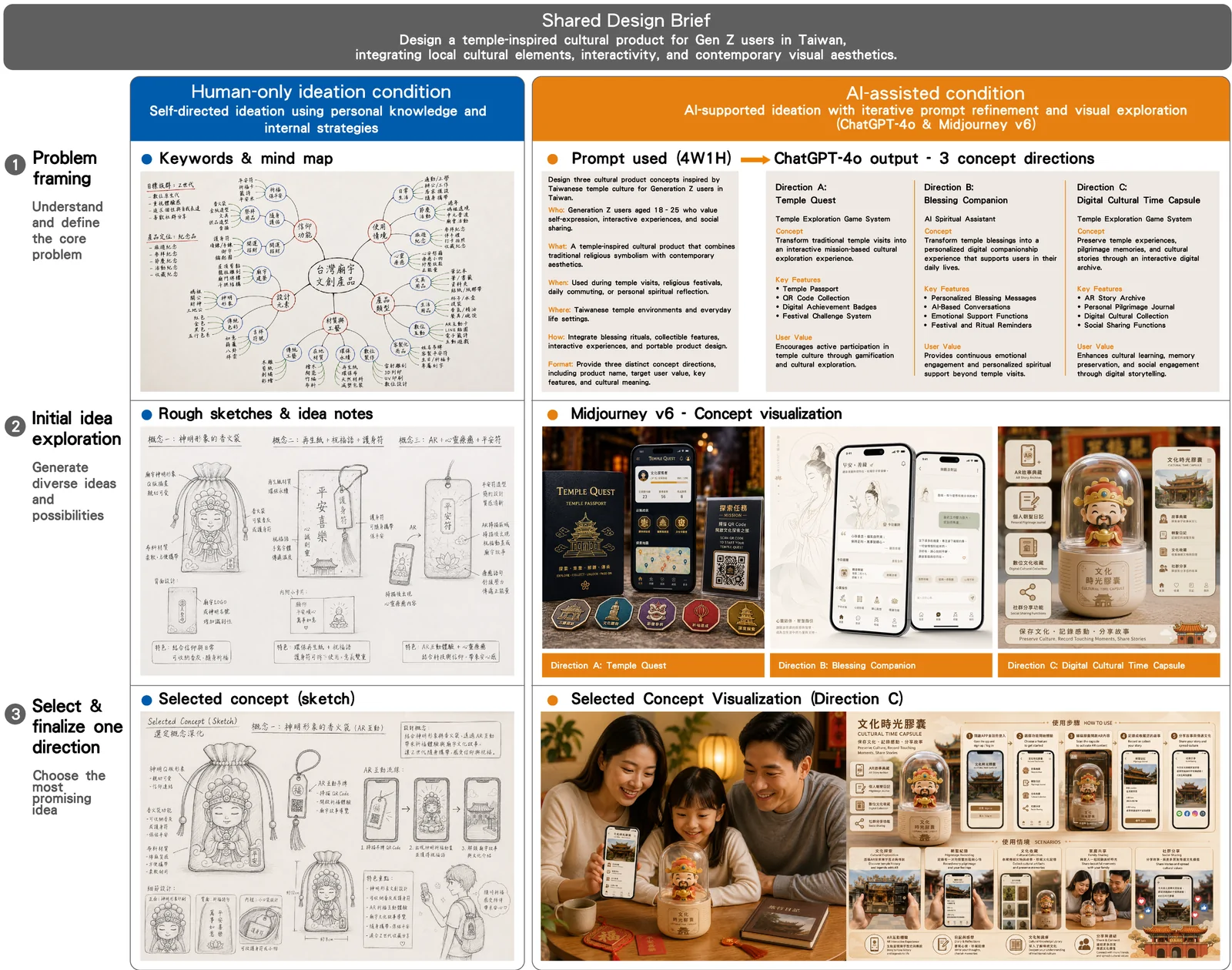
Example of the ideation-process materials and final concept development produced by the same participant under the human-only and AI-assisted conditions. The left panel shows self-directed ideation through mind mapping, keyword generation, sketching, concept selection, and refinement. The right panel shows AI-assisted ideation through ChatGPT-based concept generation and prompt refinement, Midjourney-based visual exploration, concept selection, and subsequent development. The expert raters reviewed the corresponding submitted process materials when evaluating the six ideation criteria and reviewed the final concept sheets separately when evaluating the five design attributes. The figure is presented solely as an illustrative example of the materials generated across the two workflows and was not selected to represent either high- or low-rated performance.

Formal ratings were completed independently, without discussion or consensus adjustment among the raters. No formal calibration exercise using practice cases or a consensus-rating session was conducted. Although the written instructions were intended to reduce the influence of representational differences, presentation-related expectancy effects could not be completely excluded (Fig 2).

### Rubric development

The evaluation rubrics were developed as task-specific analytic criteria grounded in prior research on design cognition, creativity evaluation, and creativity-support tools [3,11,12,39,40]. The six ideation criteria were used to differentiate observable features of early-stage ideation output, including problem structuring, exploratory breadth, comparison and justification, originality-related qualities, and translation into actionable directions. They were not proposed as latent constructs or as a general-purpose psychometric scale. In particular, creator perspective and innovation both involved originality-related judgment but differed in their operational emphasis within the rubric. Creator perspective focused on the distinctiveness and originality of the participant’s conceptualization as expressed in the proposal, whereas innovation focused on the proposal’s breakthrough potential and alignment with user or market needs. Proposed solution strategies focused on the clarity, structure, and feasibility with which a concept was translated into an actionable direction. These distinctions were operational rather than psychometric, and residual conceptual overlap cannot be excluded. The phrase “intrinsic motivation” in the original creator-perspective descriptor was not treated as a direct measure of participants’ internal motivational state; ratings were based only on observable characteristics of the submitted proposal. To preserve procedural fidelity, the scoring anchors used by the raters were not retrospectively relabeled or altered.

The five design attributes were selected to assess visible aspects of preliminary design refinement, including form, material, color, pattern, and style. These criteria were not treated as latent psychometric constructs but as structured expert-assessment categories for comparing participant-matched concept sheets. To support face and content validity, the initial rubric descriptors were reviewed by three design-domain specialists before formal evaluation. The review focused on wording clarity, conceptual distinctiveness, and applicability to undergraduate design outputs. Based on their feedback, overlapping descriptors were revised and the scoring criteria were clarified. Full scoring anchors for all five performance levels are provided in S1 and S2 Appendices. These anchors are rubric-based performance descriptors and do not reproduce or identify individual participant submissions. These anchors illustrate the intended scoring distinctions; no post hoc selection of participant outputs was used to redefine the criteria after analysis.

Because the rubrics were developed for a controlled task-specific comparison rather than for general scale development, construct validity and discriminant validity were not established through factor analysis. The study instead relied on prospective expert review of the descriptors, standardized written scoring instructions, independent ratings, and inter-rater reliability to support the credibility of the evaluation process. Criterion-level results are therefore interpreted as differences under the present analytic rubric, not as evidence of fully separable underlying creative capacities. This issue is further discussed in the Limitations and future research section.

### Subjective response questionnaire

Participants completed a task-specific post-task questionnaire developed for the present study and informed by prior work on college learning effectiveness [62]. The questionnaire was designed for condition-based comparative evaluation and was not intended to function as a standardized psychometric scale or as a validation of the original College Learning Effectiveness Inventory.

The questionnaire comprised three dimensions: perceived cognitive processing, ideation skills, and affective response (Table 3). Perceived cognitive processing assessed participants’ self-reported understanding of the task and their perceived ability to explore and organize design concepts. Ideation skills assessed perceived support for generating, extending, experimenting with, and refining design ideas. Affective response assessed participants’ enjoyment, engagement, motivation, satisfaction, and willingness to use a similar ideation approach in the future.

**Table 3:** Dimensions of the task-specific subjective-response questionnaire.

Dimension	Number of items	Construct focus
Perceived cognitive processing	5	Self-reported task understanding and concept organization
Ideation skills	5	Perceived support for idea generation, extension, experimentation, and refinement
Affective response	5	Enjoyment, engagement, motivation, satisfaction, and future-use intention

All items were written to reflect participants’ immediate experiences during the experimental tasks and were reviewed by design-domain experts for contextual relevance and wording clarity. The same item structure was administered for both conditions, with the wording referring respectively to the self-directed human-only process or the AI-assisted process. All items were rated on a 5-point Likert scale. Given its task-specific development and substantial departure from the structure of the original inventory, the questionnaire was not treated as a standardized psychometric scale. Full item wording is provided in S3 Appendix.

### Statistical analysis

All statistical analyses were conducted using IBM SPSS Statistics version 28. Descriptive statistics, including means and standard deviations, were calculated for all variables. Because the study employed a within-subjects design, paired-samples *t*-tests were used to compare the human-only and AI-assisted conditions across ideation evaluation scores, design attribute ratings, and subjective response measures. For the expert-evaluated outcomes, the three raters’ scores were averaged for each participant and evaluation criterion before the human-only and AI-assisted conditions were compared using paired-samples *t*-tests.

To control familywise Type I error, Holm–Bonferroni correction was applied separately within each defined outcome family: the six ideation criteria, the five design attributes, and the three subjective-response dimensions. Both unadjusted and Holm-adjusted *p* values are reported, and inferential interpretations are based on the adjusted values. Effect sizes for paired comparisons were reported using Cohen’s *d*_*z*_.

Inter-rater reliability was assessed using intraclass correlation coefficients based on a two-way mixed-effects, average-measures consistency model. For the ICC analyses, each rater’s scores were first averaged across the six ideation criteria or the five design attributes and then across the two experimental conditions for each participant. This procedure produced one overall participant-level score per rater for each evaluation domain and was used to estimate overall domain-level inter-rater consistency.

Exploratory sequence analyses compared the two assigned condition-order groups on participant-level condition-difference scores. These analyses examined systematic order differences but were not treated as a test that eliminates content-related carryover. Inferential interpretations for the primary outcome comparisons were based on Holm-adjusted *p* < .05 within each outcome family. The sequence-group analyses were exploratory and are reported using unadjusted two-sided *p* values.

Absolute values of Cohen’s *d*_*z*_ are reported to indicate effect magnitude; the direction of each difference is indicated by the condition means and the sign of the *t* statistic. No independent behavioral or computational measure of ideation performance was included in the original study protocol; therefore, the ICC analyses were used only to estimate inter-rater consistency and not as an objective validation of the expert ratings.

### Ethics statement

This study was reviewed by the National Taiwan Normal University Research Ethics Review Committee and was determined to qualify for exempt review (Review Category: Exempt Review; Application No. 202307ES012). The study was conducted in accordance with institutional guidelines, posed no more than minimal risk, took place within a regular educational setting, and did not involve the collection of direct personal identifiers in the analytic dataset. Before participation, participants were informed of the study purpose and procedures, the voluntary nature of participation, the coded and de-identified collection and handling of data, and their right to decline participation without academic penalty. The requirement for written informed consent was waived under the exempt-review determination, and completion of the study tasks and questionnaire was regarded as implied informed consent. All participants were undergraduate students aged 18 years or older; therefore, parental or guardian consent was not required.

## Results

### Reliability and preliminary analyses

Prior to the condition comparisons, the internal consistency of each task-specific subjective-response dimension was examined separately by experimental condition. Cronbach’s alpha values were .787 and .897 for perceived cognitive processing, .757 and .814 for ideation skills, and .877 and .919 for affective response in the human-only and AI-assisted conditions, respectively. These coefficients are reported in Table 4. Inter-rater consistency for the expert evaluations was assessed separately using intraclass correlation coefficients, as reported below.

**Table 4:** Internal consistency of the task-specific subjective-response dimensions.

Measure	Human-only condition, Cronbach’s α	AI-assisted condition, Cronbach’s α
Perceived cognitive processing	.787	.897
Ideation skills	.757	.814
Affective response	.877	.919

*Note.* Cronbach’s α values were calculated separately for each task-specific subjective-response dimension and experimental condition. The dimensions were analyzed separately and were not combined into a single overall questionnaire score.

Exploratory analyses found no statistically significant differences between the two sequence groups in participant-level condition-difference scores for any ideation-output, preliminary design-refinement, or subjective-response outcome. Mann–Whitney *U* sensitivity analyses yielded the same substantive conclusion. Detailed results are reported in S5 Appendix. These findings do not eliminate possible content-related carryover from repeated exposure to the same design brief.

### Comparison of ideation outputs between conditions (RQ1)

To address Research Question 1, paired-samples *t*-tests compared ideation-output ratings between the human-only and AI-assisted conditions. Holm–Bonferroni correction was applied across the six ideation criteria, and all six condition differences remained statistically significant after correction. The AI-assisted condition received higher ratings for problem framing (*M* = 4.64 vs. 2.94), *t*(31) = −14.03, adjusted *p* < .001, *d*_*z*_ = 2.48; mechanism (*M* = 4.48 vs. 2.99), *t*(31) = −14.08, adjusted *p* < .001, *d*_*z*_ = 2.49; creator perspective (*M* = 4.03 vs. 3.55), *t*(31) = −5.07, adjusted *p* < .001, *d*_*z*_ = 0.90; and evaluator perspective (*M* = 4.25 vs. 3.26), *t*(31) = −8.65, adjusted *p* < .001, *d*_*z*_ = 1.53. The human-only condition received higher ratings for innovation (*M* = 4.66 vs. 3.40), *t*(31) = 15.19, adjusted *p* < .001, *d*_*z*_ = 2.69, and proposed solution strategies (*M* = 4.22 vs. 3.67), *t*(31) = 8.33, adjusted *p* < .001, *d*_*z*_ = 1.47. Creator perspective and innovation should be interpreted according to their operational emphases within the task-specific rubric: the former concerned the distinctiveness and originality expressed in the proposal, whereas the latter concerned breakthrough potential and relevance to user or market needs. RQ1 was therefore answered by a differentiated profile of condition-related ratings across the six criteria (Table 5).

**Table 5:** Paired-samples *t*-test results for ideation outputs by condition.

Criterion	Human *M* (*SD*)	AI *M* (*SD*)	*t*(31)	*p*	Cohen’s *d*_*z*_	Adjusted *p* (Holm)
Problem framing	2.94 (0.571)	4.64 (0.518)	−14.032	< .001	2.48	< .001
Mechanism	2.99 (0.427)	4.48 (0.535)	−14.077	< .001	2.49	< .001
Creator perspective	3.55 (0.491)	4.03 (0.435)	−5.066	< .001	0.90	< .001
Evaluator perspective	3.26 (0.553)	4.25 (0.501)	−8.646	< .001	1.53	< .001
Innovation	4.66 (0.401)	3.40 (0.552)	15.190	< .001	2.69	< .001
Proposed solution strategies	4.22 (0.411)	3.67 (0.471)	8.330	< .001	1.47	< .001

**Fig 3:**
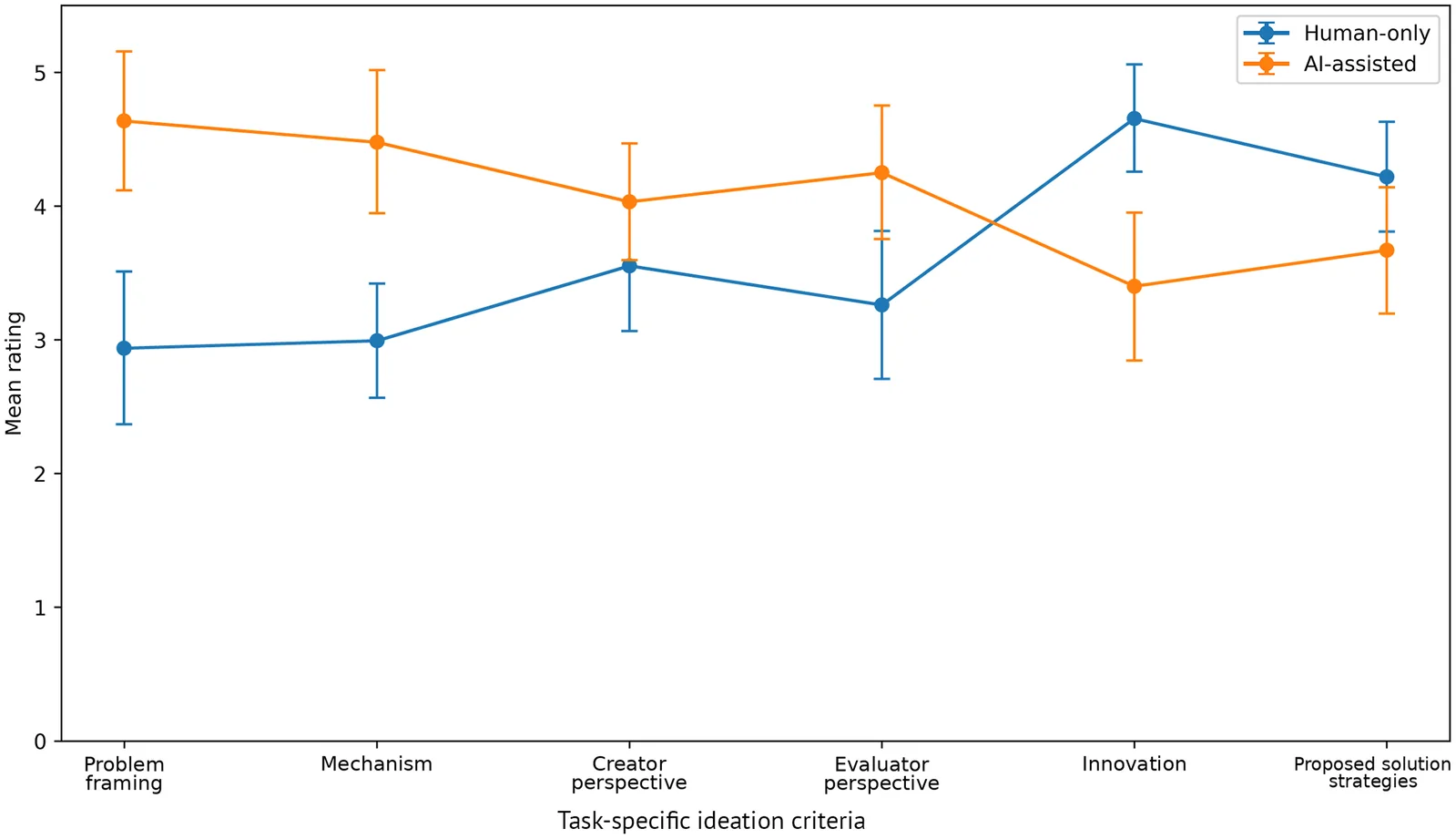
Mean ideation-output ratings across the six task-specific criteria by condition. Error bars represent ±1 standard deviation.

The mean ideation-output ratings across the six criteria are presented in Fig 3.

### Comparison of design refinement between conditions (RQ2)

To address Research Question 2, paired-samples *t*-tests were conducted to compare expert ratings of participant-matched concept sheets across five design attributes: form, material, color, pattern, and style. Across the five attributes, mean ratings were generally similar between the two conditions. No statistically significant differences were observed between the human-only and AI-assisted conditions for form (*p* = .748), material (*p* = .169), color (*p* = .294), or pattern (*p* = .205). The unadjusted comparison for style yielded *t*(31) = 2.04, *p* = .050, *d*_*z*_ = 0.36, with the human-only condition receiving slightly higher ratings than the AI-assisted condition. This comparison was not statistically significant after Holm–Bonferroni correction. Overall, these findings indicate that AI-assisted ideation did not produce a statistically robust advantage in preliminary design refinement across the evaluated attributes. RQ2 therefore yielded no statistically robust between-condition differences across the five design attributes after correction. The paired-samples *t*-test results for participant-matched concept-sheet evaluations are reported in Table 6.

**Table 6:** Paired-samples *t*-test results for participant-matched concept-sheet evaluations by condition.

Attribute	Human *M* (*SD*)	AI *M* (*SD*)	*t*(31)	*p*	Cohen’s *d*_*z*_	Adjusted *p* (Holm)
Form	4.08 (0.406)	4.11 (0.375)	−0.325	.748	0.06	.748
Material	4.26 (0.404)	4.16 (0.339)	1.408	.169	0.25	.676
Color	4.18 (0.448)	4.07 (0.336)	1.068	.294	0.19	.676
Pattern	4.18 (0.432)	4.04 (0.347)	1.294	.205	0.23	.676
Style	4.02 (0.423)	3.77 (0.467)	2.041	.050	0.36	.250

*Note.* Holm–Bonferroni correction was applied to the five design-attribute comparisons. The *p* value for style represents the uncorrected paired-samples *t*-test result.

**Fig 4:**
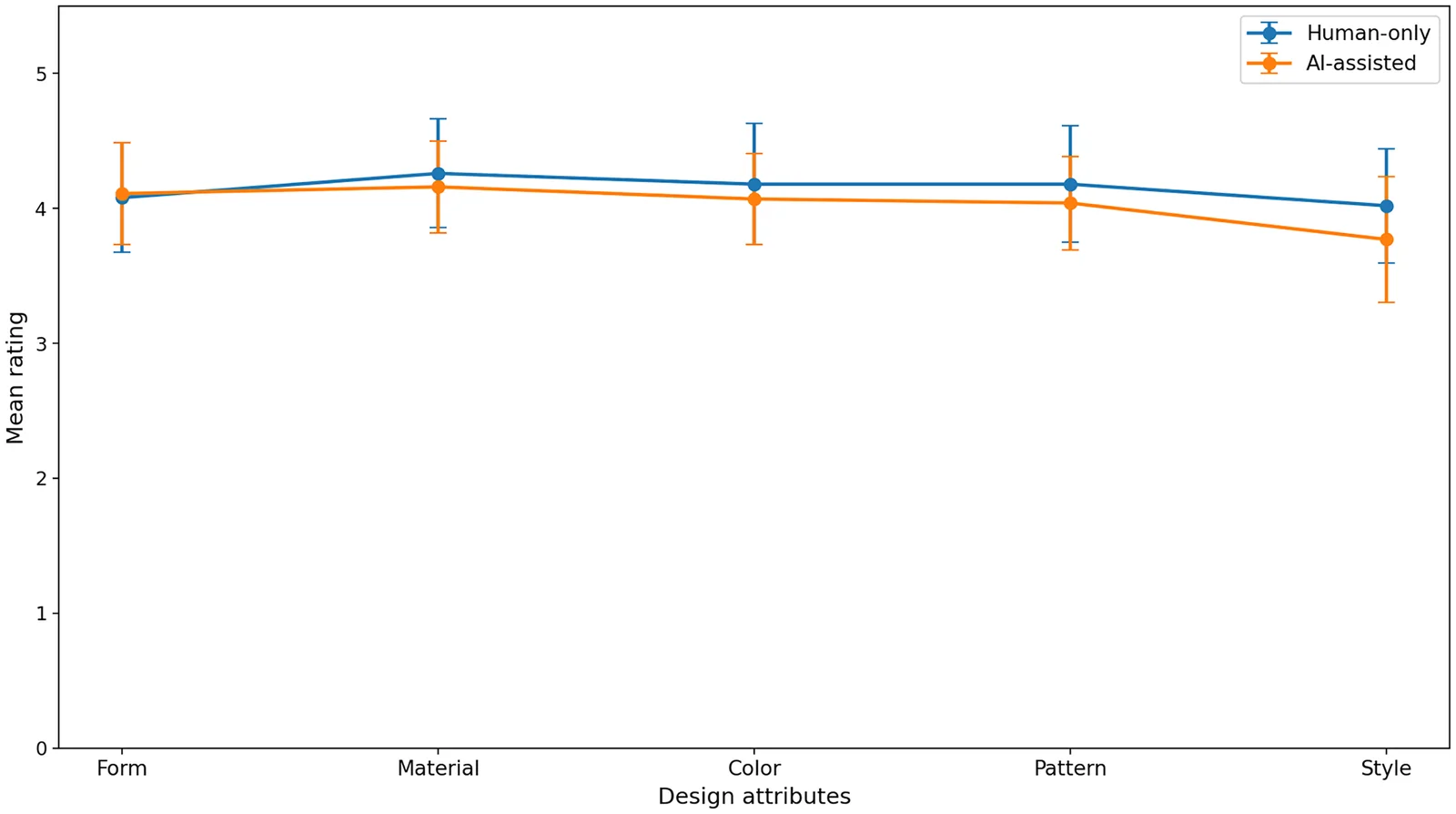
Expert ratings of participant-matched concept sheets across the five design attributes by condition. Error bars represent ±1 standard deviation.

The expert ratings of participant-matched concept sheets across the five design attributes are shown in Fig 4.

### Subjective experience comparison (H1)

To test Hypothesis 1, paired-samples *t*-tests compared subjective responses between conditions. Holm–Bonferroni correction was applied across the three subjective-response dimensions, and all three differences remained statistically significant. Perceived cognitive processing was higher in the AI-assisted condition (*M* = 4.67, *SD* = 0.47) than in the human-only condition (*M* = 4.08, *SD* = 0.44), *t*(31) = −10.23, adjusted *p* < .001, *d*_*z*_ = 1.81. Ideation skills were also higher in the AI-assisted condition (*M* = 4.44, *SD* = 0.40) than in the human-only condition (*M* = 4.06, *SD* = 0.40), *t*(31) = −7.20, adjusted *p* < .001, *d*_*z*_ = 1.27. Affective response was higher in the AI-assisted condition (*M* = 4.23, *SD* = 0.54) than in the human-only condition (*M* = 3.95, *SD* = 0.43), *t*(31) = −4.80, adjusted *p* < .001, *d*_*z*_ = 0.85. These results support Hypothesis 1 as a statement about participants’ perceived task experience; they do not by themselves demonstrate improved underlying cognition or objective design quality (Table 7).

**Table 7:** Paired-samples *t*-test results for subjective response measures by condition.

Dimension	Human *M* (*SD*)	AI *M* (*SD*)	*t*(31)	*p*	Cohen’s *d*_*z*_	Adjusted *p* (Holm)
Perceived cognitive processing	4.08 (0.437)	4.67 (0.473)	−10.225	< .001	1.81	< .001
Ideation skills	4.06 (0.404)	4.44 (0.402)	−7.198	< .001	1.27	< .001
Affective response	3.95 (0.431)	4.23 (0.527)	−4.797	< .001	0.85	< .001

**Fig 5:**
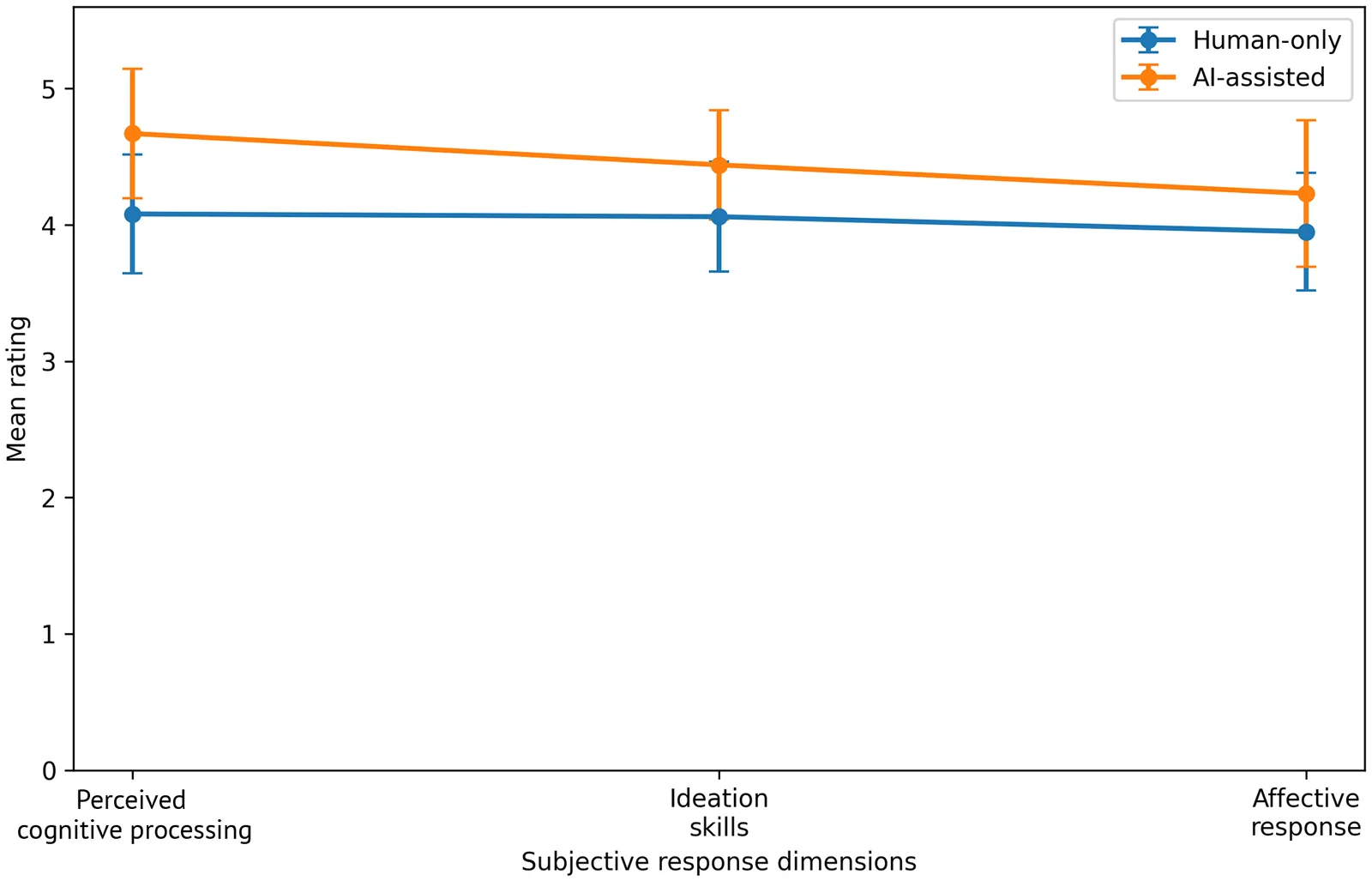
Subjective-response scores across the three measured dimensions by condition. Error bars represent ±1 standard deviation.

The subjective-response scores across the three dimensions are presented in Fig 5.

### Inter-rater reliability

Overall domain-level inter-rater reliability was assessed using the ICC procedure described in the Statistical analysis section. The average-measures ICC for ideation evaluation was .954 (95% CI [.918, .976]), indicating excellent consistency among raters. The average-measures ICC for concept-sheet evaluation was .536 (95% CI [.166, .758]). Although the point estimate indicated moderate consistency, the wide confidence interval suggested substantial uncertainty, with plausible reliability ranging from poor to good. The results therefore indicate substantially greater consistency in the evaluation of ideation outputs than in the evaluation of preliminary concept sheets. The ICC results are reported in Table 8.

**Table 8:** Inter-rater reliability for expert evaluations.

Measure	ICC	95% CI	*F*	*df*1	*df*2	*p*
Overall ideation-domain evaluation	.954	[.918, .976]	21.929	31	62	< .001
Overall concept-sheet evaluation	.536	[.166, .758]	2.156	31	62	.005

*Note.* ICCs were calculated using a two-way mixed-effects, average-measures consistency model based on participant-level scores averaged across the relevant criteria and experimental conditions.

## Discussion

### Differentiated effects of AI-assisted ideation

Previous studies have shown that generative AI can influence creative performance during design ideation. The present study adds a within-participant, multi-domain comparison of how condition-related differences were distributed across six task-specific ideation criteria and whether those differences extended to expert ratings of participant-matched concept sheets and subjective task experience. AI-assisted outputs received higher expert ratings for problem framing, mechanism, creator perspective, and evaluator perspective, whereas human-only outputs received higher ratings for innovation and proposed solution strategies. All six comparisons remained statistically significant after Holm–Bonferroni correction within the ideation outcome family. The findings therefore describe different profiles of evaluated output under a combined text-and-image AI workflow rather than a generalized advantage in creativity, cognition, or design quality.

The present findings can be differentiated from several recent studies of AI-assisted ideation. A crossover study in higher education compared AI-assisted and non-AI creative ideation [42], whereas the present study links six task-specific ideation criteria with expert ratings of participant-matched concept sheets and subjective task experience. Other research has described how generative AI may support conceptual design through problem exploration, concept generation, and representation [53]. The present experiment adds within-participant evidence that higher ratings for problem framing and mechanism can coexist with lower ratings for innovation and proposed solution strategies. Prior experimental work has also emphasized the double-edged role of generative AI in creative work [54]. Consistent with that perspective, higher perceived support and higher ratings on selected ideation criteria did not translate into a statistically robust advantage in preliminary design refinement. Exploratory breadth, perceived process support, originality-related evaluation, and downstream refinement should therefore be treated as related but non-equivalent outcomes in human–AI co-ideation.

The large effect sizes observed for problem framing and mechanism indicate substantial condition differences within this particular task and rubric. They should not be interpreted as direct evidence that AI enhanced participants’ underlying cognitive abilities. A more cautious interpretation is that the process materials and proposals produced under the combined text-and-image AI workflow displayed alternative framings, semantic associations, and explanatory relations in forms that received higher ratings under the task-specific criteria. The magnitude of these effects may also partly reflect the structured nature of the rubric, recognizable presentation characteristics of AI-assisted outputs, and the culturally specific design task used in the experiment.

Human-only outputs received higher ratings for innovation and proposed solution strategies. Under the rubric used in this study, innovation emphasized breakthrough potential and relevance to user or market needs, whereas proposed solution strategies emphasized the structured and feasible translation of concepts into actionable directions. These findings are consistent with concerns that generative systems may broaden exploration while encouraging convergence toward semantically plausible or visually familiar solutions [18,31,32]. Because cognitive processes were not directly measured, the results do not demonstrate that human-only ideation involved deeper synthesis; they indicate only that human-only outputs were rated more highly on these two task-specific criteria.

AI-assisted outputs received higher ratings for creator perspective and evaluator perspective. The creator-perspective result should be interpreted narrowly as a difference in the rated distinctiveness and originality of the conceptualization represented in the proposal; it does not demonstrate stronger authorship, intrinsic motivation, or reflective judgment. The evaluator-perspective result indicates that comparison, selection, or justification was more visible in the rated outputs, not that AI improved an underlying evaluative capacity. Because creator perspective and innovation both contained originality-related content, their contrasting condition profiles should be understood as differences between operational emphases within the present rubric rather than as evidence of fully separable psychological constructs. Possible condition recognition by raters further limits causal interpretation.

Overall, the findings support a differentiated, output-level interpretation of human–AI co-ideation rather than a generalized superiority claim.

### Limited effects on design refinement

The results for design refinement provide a more constrained interpretation of AI support. In contrast to the ideation findings, no statistically robust differences were observed between the two conditions across the five design attributes after correction for multiple comparisons. Higher ratings on selected ideation criteria in the AI-assisted condition did not translate into statistically robust differences in preliminary design refinement.

One possible explanation is that participants already possessed foundational design knowledge, which allowed them to perform at a relatively high level across both conditions. As a result, the additional contribution of AI-generated alternatives may have had limited impact on attributes such as form, color, and material, which depend on accumulated design experience and aesthetic judgment.

Another possible explanation relates to the nature of AI-mediated design refinement. In the AI-assisted condition, stylistic and visual outcomes were partially influenced by prompt formulation and system-generated outputs, which may have reduced direct control over stylistic coherence and intentional consistency. By contrast, in the human-only condition, stylistic decisions were more directly guided by participants’ internalized design knowledge and aesthetic preferences. This may relate to the slightly higher unadjusted mean rating for style in the human-only condition; however, the comparison was not statistically significant after Holm–Bonferroni correction.

The relatively moderate inter-rater reliability observed in the design refinement evaluation may also indicate that visual design assessment involves greater subjective variability across raters compared with ideation-oriented evaluation. Unlike the ideation ratings, which focused on conceptual features visible in the submitted process materials, visual-refinement judgments may depend more strongly on individual aesthetic preferences and interpretive standards.

Overall, AI-assisted outputs received higher ratings on selected ideation criteria, but these differences did not extend to statistically robust advantages in expert-rated preliminary design refinement.

### Subjective experience and perceived support

Participants rated the AI-assisted condition more positively across perceived cognitive processing, ideation skills, and affective response. These ratings indicate that the AI-assisted workflow was perceived as helping participants understand and organize the task, generate and extend ideas, and remain engaged during the activity. The immediate availability of textual and visual outputs may have made provisional alternatives easier to externalize and compare, thereby increasing perceived procedural support during ideation.

These findings should be interpreted as evidence of self-reported task experience rather than direct evidence of deeper cognitive processing or improved design ability. Participants’ positive evaluations may reflect the accessibility, responsiveness, and perceived efficiency of the AI-assisted workflow, but the study did not directly measure cognitive effort, learning, or changes in underlying creative capability. Moreover, these positive perceptions did not correspond uniformly with the expert-rated outcomes.

The subjective results therefore identify perceived support as a distinct outcome of human–AI co-ideation. In the present task, participants reported the AI-assisted process as more manageable and engaging, but these self-reported experiences did not establish stronger originality, strategic synthesis, or preliminary design refinement.

### Implications for design education and human–AI collaboration

The present findings have implications primarily for design education within the bounded context of the present task. Rather than supporting a uniform superiority claim for either condition, the results indicate different profiles of expert-rated ideation output under the human-only and AI-assisted workflows.

In the AI-assisted condition, outputs received higher ratings for problem framing, mechanism, creator perspective, and evaluator perspective, and participants reported more positive task experiences. These findings suggest potential utility for selected aspects of early-stage exploration and perceived process support. However, they should not be interpreted as evidence that AI improved participants’ underlying cognition, creativity, evaluative judgment, or learning.

Under the present task-specific rubric, human-only outputs received higher ratings on innovation and proposed solution strategies. These differences indicate that the AI-assisted workflow did not produce higher ratings on these two criteria in the present task; they do not provide direct evidence that participants possessed stronger underlying originality or strategic reasoning in the human-only condition.

The absence of statistically robust condition differences in form, material, color, pattern, and style further indicates that higher ratings on selected ideation criteria did not extend to preliminary design refinement. Perceived support, expert-rated ideation characteristics, and expert-rated refinement should therefore be treated as related but non-equivalent outcomes.

For educational practice, AI-assisted ideation may be treated as a provisional resource rather than as a substitute for independent design judgment. Learning activities could require students to compare generated alternatives, explain their selections, identify possible convergence or fixation, and independently develop and defend their final design direction. These recommendations are bounded by the present student sample, design brief, combined text-and-image workflow, and task-specific evaluation criteria.

More broadly, the findings show a differentiated distribution of expert-rated outcomes across the two conditions rather than a generalized advantage for either workflow. Because underlying cognitive operations were not directly measured, this distribution should not be interpreted as evidence that AI and human-only ideation involve distinct or complementary cognitive functions.

### Limitations and future research

This study has several limitations that should be considered when interpreting the findings. First, the sample consisted of 32 undergraduate design students from a single institution who had foundational design training but only basic prior exposure to structured AI-assisted ideation. The findings should therefore be situated within design education and should not be generalized broadly to professional design practice. In addition, the study used a single culturally situated product-design task, which limits transfer to other design domains and task types. Future research should replicate the study with larger and more diverse samples, including professional designers, interdisciplinary teams, participants with different levels of expertise, multiple institutions, and a broader range of design tasks.

Second, both conditions used the same design brief within a counterbalanced within-subjects design. Counterbalancing and the exploratory sequence analyses reduced concern about systematic order differences, but they could not eliminate content-related carryover. Participants may have reused task understanding, concepts, keywords, or visual strategies developed in the first condition when completing the second. Although the conditions were conducted on separate regular class dates, the interval was determined by the course schedule rather than implemented as a standardized washout procedure. For participants in the AI-first sequence, prior exposure to the structured AI training and prompt framework may also have influenced subsequent human-only ideation strategies. The absence of statistically significant sequence-group differences should therefore not be interpreted as evidence that carryover was absent. Future studies should preferentially use parallel but equivalent tasks or between-subject designs and, where within-subject comparisons are retained, incorporate standardized intervals and prospectively defined coding of repeated concepts or strategies across conditions.

Third, the evaluation relied on task-specific expert ratings and therefore remains subject to limitations associated with the evaluation framework and rater judgment. Although the overall domain-level ideation ICC was high, criterion-specific inter-rater reliability was not estimated, and the overall concept-sheet ICC had a moderate point estimate with a wide confidence interval, indicating greater uncertainty in the visual design evaluation. The six ideation criteria were analytically defined for the present task and were not validated as psychometrically independent constructs. In particular, creator perspective and innovation both incorporated originality-related judgment: the former emphasized the distinctiveness and originality expressed in the proposal, whereas the latter emphasized breakthrough potential and relevance to user or market needs. Residual conceptual overlap may therefore have contributed to the criterion-level pattern, and these ratings should not be interpreted as evidence of fully separable creative capacities. The phrase “intrinsic motivation” in the original creator-perspective descriptor likewise did not constitute a direct psychological measure. In addition, although raters were blinded to participant identity, they could plausibly infer experimental condition from recognizable textual and visual characteristics of the submitted materials. Presentation fidelity or expectations concerning AI-generated materials may therefore have influenced ratings independently of idea quality. The raters received standardized written instructions and complete scoring anchors, but no formal pre-rating calibration exercise using practice cases was conducted. Future research should refine the criteria prospectively, examine their conceptual distinctiveness through independent expert review or rater cognitive interviews, incorporate calibration before formal scoring, standardize representational fidelity more strictly, conduct condition-recognition checks, and include additional independent raters.

Fourth, the AI-assisted condition combined ChatGPT-4o and Midjourney v6 within a single multimodal workflow. The study therefore cannot determine whether the observed differences arose from language-based reframing, image-based stimulation, or interactions between the two forms of support. Representative prompts, tool-role descriptions, and process records improve procedural transparency but do not permit separate causal attribution to the two systems. Complete standardized tool-specific interaction logs were also not available for process-level analysis. Future factorial or staged experiments should compare text-only, image-only, and combined AI conditions while collecting complete interaction logs and predefined process indicators. Because generative AI systems are stochastic and may change over time, reproducibility of the present experiment should be understood primarily in procedural terms rather than as exact regeneration of identical textual or visual outputs.

Fifth, the study examined short-term task performance and participants’ immediate subjective experience rather than longer-term learning, dependence, changes in design judgment, or downstream implementation quality. The task-specific questionnaire was developed for condition-based comparison and was not validated as a general psychological scale; consequently, higher self-reported support should not be interpreted as evidence of deeper cognitive processing or objectively superior design performance. Participant-level ideation processes were also not systematically captured through screen recording, think-aloud protocols, or complete real-time process logs, and some positive subjective responses may have reflected novelty effects. Moreover, the original protocol did not include an independent behavioral, computational, or performance-based indicator with which to triangulate the expert ratings. Inter-rater reliability assesses consistency among raters but does not independently validate the rated criteria, while the questionnaire provides a complementary self-reported perspective rather than objective evidence of creative performance. The present findings should therefore be interpreted as convergence or divergence across expert-rated outputs and participant-reported experience, rather than as objective triangulation of creative performance. Future studies should prospectively combine expert evaluation with preregistered indicators such as the number of distinct concept directions, conceptual-category or semantic diversity, iteration counts, cross-condition concept overlap, implementation feasibility, user evaluation, and other behavioral or computational process measures, and should examine whether short-term perceived support translates into sustained learning or design capability over time.

Finally, future research could extend this human–AI co-ideation framework to design tasks explicitly oriented toward the Sustainable Development Goals (SDGs). In design education, subsequent studies could examine whether AI-supported ideation helps students frame sustainability problems, explore alternatives, and justify responsible design decisions, with particular relevance to SDG 4 (Quality Education). Sustainability-oriented briefs involving resource efficiency, circular product development, or socially responsive design could also be used to examine potential relevance to SDG 9 (Industry, Innovation and Infrastructure) and SDG 12 (Responsible Consumption and Production). Importantly, the present study did not measure sustainability outcomes; these connections should therefore be treated as directions for future empirical evaluation rather than as benefits established by the current findings. Future studies should incorporate explicit sustainability criteria and downstream indicators to determine whether AI-supported ideation translates into more sustainable design outcomes.

### Contributions

Theoretically, the contribution of this study does not lie in proposing new ideation constructs or in claiming that generative AI has uniformly or selectively beneficial effects. Rather, it lies in showing, within the same counterbalanced experiment, that task-specific expert ratings of ideation output, expert ratings of participant-matched concept sheets, and participants’ perceived task experience can exhibit different condition-related profiles. This distinction extends prior accounts of selective AI effects by showing, within the same experiment, that perceived process support and higher ratings on selected structuring criteria can coexist with lower ratings on innovation and proposed solution strategies and with no robust difference in preliminary design refinement.

Methodologically, the additional contribution is the linked within-participant comparison of three non-equivalent outcome domains: multi-criterion expert ratings of ideation outputs, expert ratings of participant-matched concept sheets, and participant-reported task experience. This design enables direct examination of whether condition-related differences at one stage or level of early design work are reproduced at another. The task-specific criteria are used transparently as analytic categories rather than presented as a newly validated psychometric instrument, and inferential interpretations are based on familywise multiple-comparison correction within each outcome family.

Practically, the study supports a bounded and stage-sensitive approach to generative AI in design education. AI may be used to externalize alternatives, reframe an open-ended brief, and support comparison during exploratory work, while originality, strategic synthesis, and final evaluative responsibility remain explicit responsibilities of the learner. Educational activities should therefore require students not only to generate with AI but also to justify selections, identify possible convergence or fixation, and independently develop and defend their final design direction (Table 9).

**Table 9:** Summary of comparative findings across outcome domains.

Outcome domain	Higher-rated outcomes in the AI-assisted condition	Higher-rated outcomes in the human-only condition	Interpretatio*n*
Ideation output	Problem framing, mechanism, creator perspective, and evaluator perspective	Innovation and proposed solution strategies	The two conditions produced different profiles of expert-rated ideation output rather than a uniform advantage for either condition.
Design refinement	None after Holm–Bonferroni correction	None after Holm–Bonferroni correction	Condition-related differences in ideation did not extend to robust differences in preliminary design refinement.
Subjective experience	Perceived cognitive processing, ideation skills, and affective response	None	Participants perceived the AI-assisted workflow as more supportive, but these perceptions did not parallel the expert-rated outcomes.

*Note.* Higher ratings indicate condition-related differences within the present task and evaluation framework and should not be interpreted as evidence of superior underlying cognitive ability.

## Conclusion

By considering task-specific ideation-output ratings, expert ratings of participant-matched concept sheets, and subjective task experience within the same experiment, the study provides a differentiated account of AI-assisted design activity beyond an aggregate comparison of creativity or output quality.

The findings indicate that the two conditions produced different profiles of expert-rated ideation output. These differences did not extend to a statistically robust advantage in preliminary design refinement, although participants perceived the AI-assisted process as more supportive and engaging. The three outcome domains therefore did not vary in parallel.

AI may assist the externalization and comparison of provisional ideas, but perceived support and selected output-level ratings should not be treated as evidence of stronger underlying cognition, authorship, originality, or downstream design quality. Within the limits of the present sample and experimental design, generative AI is most appropriately positioned as scaffolding for exploratory work while independent judgment and evaluative responsibility remain with the designer.

## Supporting information

S1 Appendix Task-specific analytic rubric and scoring anchors for ideation evaluation across six analytic criteria. (DOCX)

S2 Appendix Full rubric for participant-matched concept-sheet evaluation across five design attributes. (DOCX)

S3 Appendix Items of the task-specific subjective-response questionnaire. (DOCX)

S4 Appendix Experimental task, stage-specific instructions, submission requirements, tool-role mapping, and illustrative prompt-refinement sequence. (DOCX)

S5 Appendix Exploratory sequence analyses for condition-order effects. (DOCX)

S1 Dataset Anonymized participant-level dataset containing rater-level expert evaluation scores and item-level subjective-response data for the human-only and AI-assisted conditions, together with sequence-assignment information used to reproduce the reported outcome descriptive statistics, reliability analyses, and inferential tests. (XLSX)
