# Supplementary material for: Generative AI as a cognitive support tool in early-stage design ideation: A human–AI co-ideation experiment
Source: PLoS One. 2026 Sep 17;21(9):e0358542. doi: 10.1371/journal.pone.0358542 (PMC13585246; doi:10.1371/journal.pone.0358542)
Supplement: Supplementary file 1 [file pone.0358542.s001.docx]

**S1 Appendix. Task-specific analytic rubric and scoring anchors for ideation evaluation across six analytic criteria.**

| Criterion | Definition | Excellent (5) | Good (4) | Fair (3) | Poor (2) | Very Poor (1) |
| --- | --- | --- | --- | --- | --- | --- |
| Problem framing | The extent to which the core design problem is clearly defined and focused | The problem is clearly defined and effectively aligned with real-world needs (e.g., local or industry-specific issues) | The problem is generally clear with some connection to real-world needs | The problem is partially defined but lacks focus or relevance | The problem is vague and weakly connected to real-world needs | The problem is unclear or not identifiable |
| Mechanism | The ability to generate diverse ideas and move beyond conventional thinking frameworks | Idea generation is highly diverse, demonstrating cross-domain perspectives and innovative thinking pathways | Ideas show some diversity and occasional novel perspectives | Ideas are somewhat varied but remain within familiar frameworks | Limited diversity in ideas, mostly conventional | Ideas are repetitive and lack variation |
| Creator perspective | Originality and intrinsic motivation of creative ideas | Ideas demonstrate strong originality and novelty, with distinctive conceptualization | Ideas show moderate originality with some novel elements | Ideas show limited originality and rely on familiar concepts | Ideas are mostly derivative with minimal novelty | Ideas lack originality and are highly conventional |
| Evaluator perspective | Ability to select, compare, and refine ideas effectively | Ideas are systematically compared and integrated with clear strategic and logical reasoning | Ideas are compared with some logical justification | Limited comparison of ideas with weak justification | Minimal evaluation or comparison of ideas | No clear evaluation or selection process |
| Innovation | The extent to which ideas demonstrate breakthrough potential | Ideas show clear innovation and strong alignment with user or market needs | Ideas demonstrate some innovative elements with partial relevance to needs | Ideas contain limited innovation | Ideas show minimal innovation | No evidence of innovation |
| Proposed solution strategies | Ability to translate ideas into concrete design proposals | Proposals are clearly structured, with a comprehensive and feasible design process | Proposals are generally clear with identifiable structure | Proposals are partially developed but lack clarity or completeness | Proposals are vague and underdeveloped | No clear proposal or implementation strategy |

*Note.* The wording shown reproduces the rubric provided to the raters. Although the original descriptor included “intrinsic motivation,” no internal motivational state was directly measured. Ratings were operationally based on the observable distinctiveness and originality described in the scoring anchors.
