# Supplementary material for: Generative AI as a cognitive support tool in early-stage design ideation: A human–AI co-ideation experiment
Source: PLoS One. 2026 Sep 17;21(9):e0358542. doi: 10.1371/journal.pone.0358542 (PMC13585246; doi:10.1371/journal.pone.0358542)
Supplement: Supplementary file 2 [file pone.0358542.s002.docx]

**S2 Appendix. Full rubric for participant-matched concept-sheet evaluation across five design attributes.**

| Attribute | Definition | Excellent (5) | Good (4) | Fair (3) | Poor (2) | Very Poor (1) |
| --- | --- | --- | --- | --- | --- | --- |
| Form | Clarity and creativity of the overall form, including structural coherence, fluency of lines, proportional balance, and consistency with aesthetic and functional requirements | The form is highly coherent and creative, with fluent lines, balanced proportions, and strong consistency with both aesthetic and functional requirements | The form is generally coherent, with minor weaknesses in proportion, line quality, or functional consistency | The form is acceptable but only moderately coherent or refined | The form is weakly organized, with limited coherence or aesthetic control | The form is inappropriate, unclear, or poorly resolved |
| Material | Appropriateness of material selection for the product concept, including suitability, expressiveness, and contribution to design quality | Material selection is highly appropriate, strengthens the design expression, and demonstrates excellent perceived quality | Material selection is generally appropriate and supports the design | Material selection is acceptable but only moderately appropriate | Material selection is weak and only partially supports the design | Material selection is inappropriate or poorly considered |
| Color | Quality of color application in terms of innovation, thematic fit, and aesthetic integration | The color scheme is highly integrated with the product theme and demonstrates strong aesthetic quality | The color scheme is generally well integrated with the theme | The color scheme is acceptable but only moderately integrated | The color scheme shows weak integration with the theme | The color scheme is insufficient or inappropriate |
| Pattern | Degree to which the pattern design is innovative, visually engaging, and consistent with the product theme | The pattern design is highly consistent with the product theme, visually refined, and strongly engaging | The pattern design is generally consistent with the theme, with acceptable visual treatment | The pattern design is adequate but only moderately connected to the theme | The pattern design is weakly connected to the theme and lacks visual refinement | The pattern design does not fit the theme |
| Style | Clarity, consistency, and recognizability of the overall design style in communicating the design intention | The overall style is highly consistent, distinctive, and clearly communicates the design intention | The overall style is generally consistent and recognizable | The overall style is acceptable but only moderately clear or consistent | The overall style is weak and lacks coherence | The overall style is unclear or not recognizable |
