# Supplementary material for: Generative AI as a cognitive support tool in early-stage design ideation: A human–AI co-ideation experiment
Source: PLoS One. 2026 Sep 17;21(9):e0358542. doi: 10.1371/journal.pone.0358542 (PMC13585246; doi:10.1371/journal.pone.0358542)
Supplement: Supplementary file 3 [file pone.0358542.s003.docx]

**S3 Appendix. Items of the task-specific subjective-response questionnaire.**

| Dimension | Item |
| --- | --- |
| Perceived cognitive processing | 1. The ideation process in this condition helped me understand the core problem of the design task more clearly. |
|  | 2. The ideation process in this condition helped me explore multiple possible design directions. |
|  | 3. The ideation process in this condition supported the development of more structured design concepts. |
|  | 4. The ideation process in this condition helped me generate ideas more efficiently. |
|  | 5. The ideation process in this condition encouraged deeper thinking about the design task. |
| Ideation skills | 1. The ideation process in this condition helped me develop more creative ideas. |
|  | 2. The ideation process in this condition improved my ability to expand design concepts. |
|  | 3. The ideation process in this condition helped me refine and organize design ideas more effectively. |
|  | 4. The ideation process in this condition supported experimentation with different concept possibilities. |
|  | 5. The ideation process in this condition enhanced my confidence during concept development. |
| Affective response | 1. I found the ideation process in this condition enjoyable. |
|  | 2. I felt engaged during the ideation process in this condition. |
|  | 3. The ideation process in this condition increased my motivation to continue developing the concept. |
|  | 4. I was satisfied with the overall ideation experience in this condition. |
|  | 5. I would be willing to use a similar ideation approach again in future design tasks. |

*Notes*.

- The same questionnaire structure was used for both conditions.
- In the human-only condition, items referred to participants’ self-directed ideation process without AI assistance.
- In the AI-assisted condition, items referred to the ideation process supported by ChatGPT-4o and Midjourney v6.
- The questionnaire was developed for the present study for condition-based comparative evaluation and was informed by prior work on college learning effectiveness. Given its task-specific development, it was not intended to function as a standardized psychometric scale.
