# Supplementary material for: Generative AI as a cognitive support tool in early-stage design ideation: A human–AI co-ideation experiment
Source: PLoS One. 2026 Sep 17;21(9):e0358542. doi: 10.1371/journal.pone.0358542 (PMC13585246; doi:10.1371/journal.pone.0358542)
Supplement: Supplementary file 4 [file pone.0358542.s004.docx]

**S4 Appendix. Experimental task, stage-specific instructions, submission requirements, tool-role mapping, and illustrative prompt-refinement sequence.**

**1. Shared design brief**

Design a temple-inspired cultural product for Gen Z users in Taiwan, integrating local cultural elements, interactivity, and contemporary visual aesthetics. The same design brief was used in both the human-only and AI-assisted conditions. Participants were asked to identify relevant user and cultural considerations, generate multiple concept directions, and select one direction for further development.

**2. Session arrangement**

The two experimental conditions were completed on separate dates. Each condition followed the same three-stage task structure: (1) problem framing, (2) initial idea exploration, and (3) selection and finalization of one concept direction. Each stage lasted 20 minutes, resulting in a total ideation duration of 60 minutes per condition. Following the 60-minute ideation task, participants assembled the selected concept into a standardized concept sheet. No separate standardized time limit was imposed for concept-sheet assembly; however, the instructions and required information structure were identical across conditions. Immediately after completing the concept-sheet assembly, participants completed the task-specific subjective-response questionnaire for that condition.

**3. Stage-specific task structure**

| Stage | Duration | Shared task objective | Human-only condition | AI-assisted condition |
| --- | --- | --- | --- | --- |
| Problem framing | 20 min | Interpret the design brief and identify the core problem, users, contextual requirements, and possible directions | Participants used keywords, mind maps, written notes, and their own prior knowledge | Participants used the shared brief and structured prompts with ChatGPT-4o to explore users, context, requirements, and possible directions |
| Initial idea exploration | 20 min | Generate and examine multiple concept possibilities | Participants produced rough sketches, idea notes, and self-generated alternatives | Participants iteratively used ChatGPT-4o for textual expansion and Midjourney v6 for visual exploration |
| Selection and finalization | 20 min | Compare the alternatives, select one promising direction, and articulate the selected concept | Participants selected and refined one direction using sketches and written explanation | Participants selected one direction and further developed it using textual and visual materials generated during the preceding stages |

*Note.* The design brief, stage sequence, time allocation, classroom environment, and conceptual output requirements were held constant across conditions. The principal experimental difference was the availability of ChatGPT-4o and Midjourney v6 in the AI-assisted condition. The representational formats were not identical because the human-only condition relied primarily on participant-generated sketches and written notes, whereas the AI-assisted condition could include language-model outputs and generated imagery.

**4. Condition-specific instructions**

**Human-only condition**

Participants completed all three stages without generative AI tools or other external idea-generation systems. They worked individually and relied on their own design knowledge, experience, associative thinking, sketching, keyword generation, and self-directed ideation strategies. Internet-based idea-generation systems and AI-generated textual or visual materials were not permitted. Participants documented their problem interpretation, alternative concepts, comparison process, and selected design direction in the submitted task materials.

**AI-assisted condition**

Participants completed the same three stages using ChatGPT-4o and Midjourney v6 as ideation-support tools. Prompt iteration was unrestricted throughout the three stages. No fixed Midjourney style presets, API settings, temperature adjustments, or researcher-imposed output-selection rules were applied. Participants remained responsible for revising prompts, comparing generated alternatives, rejecting unsuitable outputs, and selecting the concept direction they considered most appropriate.

No new AI-generated content was produced during the subsequent concept-sheet assembly stage. However, Midjourney-generated images produced during the 60-minute ideation process could be retained as visual representations in the final concept sheets.

**5. Tool-role mapping across the three-stage workflow and subsequent concept-sheet assembly**

| Stage | Human-only activity | ChatGPT-4o role | Midjourney v6 role | Participant responsibility | Required evidence |
| --- | --- | --- | --- | --- | --- |
| Problem framing 20 min | Interpret the brief using keywords, mind maps, written notes, and prior design knowledge | Interpret and reframe the brief; generate textual concept directions | Generally not required at this stage, although preliminary visual exploration was permitted | Evaluate the relevance of suggested framings and determine which directions warranted further exploration | Problem-framing notes, keywords, mind maps, prompts, or textual exploration records |
| Initial idea exploration 20 min | Generate multiple alternatives through sketching, associative thinking, and self-generated concept notes | Expand concepts and refine textual prompts | Generate preliminary visual alternatives from selected textual directions | Compare alternatives, revise prompts, reject unsuitable outputs, and identify promising concepts | Multiple concept directions, sketches, textual outputs, prompts, and generated visual alternatives |
| Selection and finalization 20 min | Compare alternatives, select one direction, and refine it through sketches and written explanation | Further articulate the selected concept and refine prompts as needed | Refine or regenerate visual alternatives for the selected direction | Select, integrate, and further articulate one concept proposal | Selected concept, rationale, supporting sketches or annotations, and relevant generated visual materials |
| Concept-sheet assembly Subsequent stage | Assemble the selected concept into the standardized layout | No new text generation permitted | No new image generation permitted | Organize the selected title, description, and visual representation into the concept sheet | Final standardized concept sheet |

*Note.* The table describes the intended functions of the two AI tools within the experimental workflow. It does not indicate that all participants used the tools in an identical order or with the same number of iterations. Because ChatGPT-4o and Midjourney v6 were incorporated within one combined multimodal condition, the study does not permit separate causal attribution of the observed outcomes to textual reframing, visual stimulation, or their interaction.

**6. 4W1H-based prompt framework with an additional output-format specification**

Participants were introduced to a 4W1H-based prompt framework with an additional output-format specification. The framework was intended to support contextual clarity while allowing participants to revise, expand, or depart from the example wording according to their own design directions.

| Prompt Element | Description |
| --- | --- |
| Who | Gen Z users aged 18–25 |
| What | Temple-inspired cultural product |
| When | Temple festival season |
| Where | Taiwan, particularly an urban temple context |
| How | Interactive, culturally engaging, and visually contemporary design |
| Format | Preliminary concept directions and visual representations |

**7. Illustrative prompt-refinement sequence**

| Iteration | Prompt Function | Tool | Example Prompt |
| --- | --- | --- | --- |
| 1 | Problem Framing | ChatGPT-4o | Generate three design directions for a temple-inspired cultural product targeting Gen Z users in Taiwan during temple festival season. The concept should integrate local cultural symbols, interactive elements, and contemporary visual aesthetics. |
| 2 | Concept Expansion | ChatGPT-4o | Develop the second concept into a temple-inspired cultural product using symbolic temple imagery, sustainable materials, modular structure, and interactive user experience features. |
| 3 | Visual Exploration | Midjourney v6 | Generate preliminary concept visualizations for a temple-inspired cultural product using folk-inspired colors, contemporary styling, and collectible or interactive design elements. |

*Note.* The following prompts illustrate the intended use of the structured prompt framework. They do not represent complete participant interaction logs and were not used as fixed prompts that all participants were required to reproduce verbatim.

**8. Submission and evaluation materials**

For each condition, participants submitted the available materials produced across the three task stages, including problem-framing records, alternative-concept materials, selection and refinement records, and the selected final concept. The expert raters reviewed these submitted process materials when applying the six ideation criteria.

Participants also assembled one selected concept from each condition into a common concept-sheet layout containing a title, a brief concept description, and a visual representation. These concept sheets were evaluated separately across form, material, color, pattern, and style.

Participant identifiers were removed before evaluation. However, because the original representational characteristics of the materials were retained, the evaluation was blinded to participant identity but not fully blinded to experimental condition.
