# Supplementary material for: Generative AI as a cognitive support tool in early-stage design ideation: A human–AI co-ideation experiment
Source: PLoS One. 2026 Sep 17;21(9):e0358542. doi: 10.1371/journal.pone.0358542 (PMC13585246; doi:10.1371/journal.pone.0358542)
Supplement: Supplementary file 5 [file pone.0358542.s005.docx]

**S5 Appendix. Exploratory sequence analyses for condition-order effects.**

Participants were assigned to one of two counterbalanced sequences. Participants P01–P16 completed the human-only condition first and the AI-assisted condition second (human-first sequence; *n* = 16), whereas participants P17–P32 completed the AI-assisted condition first and the human-only condition second (AI-first sequence; *n* = 16).

For each outcome, a participant-level condition-difference score was calculated by subtracting the human-only score from the AI-assisted score. Positive values therefore indicate higher scores in the AI-assisted condition relative to the human-only condition, whereas negative values indicate higher scores in the human-only condition. For the expert-evaluated outcomes, ratings were first averaged across the three raters for each participant and evaluation criterion.

The condition-difference scores were compared between the two sequence groups using independent-samples *t*-tests. Normality within each sequence group was examined using Shapiro–Wilk tests, and homogeneity of variance was assessed using Levene’s tests. Because several difference-score distributions departed from normality, two-sided Mann–Whitney *U* tests were additionally conducted as sensitivity analyses. Welch’s *t*-test was used for affective response because the homogeneity-of-variance assumption was not met. Cohen’s *d* values represent the standardized difference between the human-first and AI-first sequence groups.

**Table S5.1. Assumption diagnostics for participant-level condition-difference scores.**

| **Outcome** | **Shapiro–Wilk *p*, human-first** | **Shapiro–Wilk *p*, AI-first** | **Levene’s test *p*** |
| --- | --- | --- | --- |
| **Ideation output** | | | |
| Problem framing | .121 | .298 | .205 |
| Mechanism | .347 | .013 | .433 |
| Creator perspective | .033 | .022 | .226 |
| Evaluator perspective | .688 | .075 | .295 |
| Innovation | .029 | .217 | .297 |
| Proposed solution strategies | .069 | .255 | .889 |
| **Preliminary design refinement** | | | |
| Form | .515 | .811 | .403 |
| Material | .090 | .090 | .291 |
| Color | .524 | .152 | .934 |
| Pattern | .299 | .558 | .230 |
| Style | .049 | .900 | .602 |
| **Subjective task experience** | | | |
| Perceived cognitive processing | .001 | .021 | .731 |
| Ideation skills | .251 | .016 | .611 |
| Affective response | .001 | .012 | .001 |

*Note.* Values below .05 indicate evidence of departure from normality or homogeneity of variance. The variance assumption was met for all outcomes except affective response. Because several Shapiro–Wilk tests were significant, Mann–Whitney *U* sensitivity analyses were conducted for all outcomes.

**Table S5.2. Exploratory comparisons of condition-difference scores between sequence groups.**

Panel A. Ideation output

| **Outcome** | **Human-first *M* (*SD*)** | **AI-first *M* (*SD*)** | ***t* (*df*)** | ***p*** | **Cohen’s *d*** |
| --- | --- | --- | --- | --- | --- |
| Problem framing | 1.729 (0.752) | 1.667 (0.632) | 0.254 (30) | .801 | 0.090 |
| Mechanism | 1.417 (0.638) | 1.563 (0.567) | −0.683 (30) | .500 | −0.242 |
| Creator perspective | 0.562 (0.580) | 0.396 (0.490) | 0.878 (30) | .387 | 0.310 |
| Evaluator perspective | 1.000 (0.750) | 0.979 (0.551) | 0.090 (30) | .929 | 0.032 |
| Innovation | −1.354 (0.412) | −1.167 (0.516) | −1.135 (30) | .265 | −0.401 |
| Proposed solution strategies | −0.521 (0.384) | −0.583 (0.375) | 0.466 (30) | .645 | 0.165 |

Panel B. Preliminary design refinement

| **Outcome** | **Human-first *M* (*SD*)** | **AI-first *M* (*SD*)** | ***t* (*df*)** | ***p*** | **Cohen’s *d*** |
| --- | --- | --- | --- | --- | --- |
| Form | 0.021 (0.590) | 0.042 (0.515) | −0.106 (30) | .916 | −0.038 |
| Material | −0.167 (0.365) | −0.042 (0.469) | −0.841 (30) | .407 | −0.297 |
| Color | −0.229 (0.540) | 0.021 (0.551) | −1.296 (30) | .205 | −0.458 |
| Pattern | −0.083 (0.524) | −0.188 (0.666) | 0.492 (30) | .627 | 0.174 |
| Style | −0.188 (0.720) | −0.313 (0.683) | 0.504 (30) | .618 | 0.178 |

Panel C. Subjective task experience

| **Outcome** | **Human-first *M* (*SD*)** | **AI-first *M* (*SD*)** | ***t* (*df*)** | ***p*** | **Cohen’s *d*** |
| --- | --- | --- | --- | --- | --- |
| Perceived cognitive processing | 0.625 (0.386) | 0.550 (0.258) | 0.646 (30) | .523 | 0.229 |
| Ideation skills | 0.450 (0.306) | 0.325 (0.300) | 1.168 (30) | .252 | 0.413 |
| Affective response | 0.338 (0.405) | 0.213 (0.213) | 1.094 (22.69)† | .286 | 0.387 |

*Note.* Condition-difference scores were calculated as AI-assisted minus human-only. Positive scores indicate relatively higher scores under AI assistance, whereas negative scores indicate relatively higher scores under the human-only condition. Cohen’s *d* was calculated as the standardized difference between the human-first and AI-first sequence groups; positive values indicate a larger AI-minus-human difference in the human-first sequence. † Welch’s *t*-test was used because Levene’s test indicated unequal variances.

**Table S5.3. Mann–Whitney *U* sensitivity analyses of condition-difference scores by sequence group.**

| **Outcome** | **Mann–Whitney *U*** | **Two-sided *p*** |
| --- | --- | --- |
| **Ideation output** | | |
| Problem framing | 139.5 | .677 |
| Mechanism | 100.5 | .305 |
| Creator perspective | 152.0 | .366 |
| Evaluator perspective | 132.0 | .895 |
| Innovation | 97.0 | .242 |
| Proposed solution strategies | 147.0 | .480 |
| **Preliminary design refinement** | | |
| Form | 131.0 | .924 |
| Material | 112.5 | .564 |
| Color | 89.5 | .147 |
| Pattern | 139.0 | .688 |
| Style | 138.0 | .719 |
| **Subjective task experience** | | |
| Perceived cognitive processing | 132.0 | .894 |
| Ideation skills | 159.5 | .237 |
| Affective response | 139.0 | .678 |

*Note.* All tests were two-sided and used asymptotic significance with continuity correction. The Mann–Whitney analyses produced the same substantive conclusion as the independent-samples and Welch’s *t*-tests.

**Interpretation**

No statistically significant differences were detected between the human-first and AI-first sequence groups for any ideation-output, preliminary design-refinement, or subjective-response difference score. The smallest parametric *p* value was .205, and all Mann–Whitney sensitivity analyses were also non-significant, with two-sided *p* values of .147 or greater. The conclusions were therefore unchanged when departures from normality and unequal variance in the affective-response outcome were taken into account.

These analyses provide no evidence of systematic differences in condition effects between the two assigned sequences. However, the absence of statistically significant sequence-group differences should not be interpreted as evidence that carryover was eliminated. With only 16 participants in each sequence group, the analyses had limited sensitivity to smaller order-related effects. In addition, ideas, task understanding, keywords, or visual strategies acquired during the first condition could have transferred to the second condition without producing a detectable between-sequence difference.
